# The Role of Machine Learning in Management of Operating Room: A Systematic Review

**DOI:** 10.7759/cureus.79400

**Published:** 2025-02-21

**Authors:** Alaa Merghani Abdelrazig Merghani, Abdullah Khaled Ahmed Esmail, Ahmed Mohamed Elamin Mubarak Osman, Nihal Ahmed Abdelfrag Mohamed, Safwa Mustafa Mohamed Ali Shentour, Shaima Merghani Abdelrazig Merghani

**Affiliations:** 1 General Medicine, National Ribat University, Khartoum, SDN; 2 Surgery, Dubai Academic Health Corporation, Dubai, ARE; 3 Clinical Sciences, Sulaiman Alrajhi University, Albukairiah, SAU; 4 General Medicine, Jouf University Medical Services Center, Sakaka, SAU; 5 Obstetrics and Gynecology, Armed Forces Hospital, Ministry of Defense Health Services, Wadi AlDawsir, SAU; 6 Emergency Medicine, National Ribat University, Khartoum, SDN

**Keywords:** artificial intelligence, machine learning, operating room, operation theatre, surgery

## Abstract

Machine learning (ML) is a developing technology that enables the analysis and interpretation of large amounts of data. The purpose of this systematic review was to summarize the available literature on the role of ML in operating room (OR) management. The Preferred Reporting Items for Systematic Reviews and Meta-Analyses (PRISMA) guidelines were followed to search the literature based on pre-defined inclusion and exclusion criteria. A total of 608 studies were found across five different databases (PubMed, EMBASE, Scopus, Web of Science, and IEEE Xplore), of which 21 studies were included in this review after removing duplicates and excluding studies based on the pre-defined inclusion and exclusion criteria. The review highlights how ML has a major impact on surgical case cancellation detection, post-anesthesia unit resource allocation optimization, and surgical case length prediction. Neural networks, XGBoost, and random forests are a few examples of ML algorithms that have shown promise in increasing prediction accuracy and resource efficiency. Nonetheless, issues including privacy concerns and data access remain challenges. The study emphasizes how ML is advancing in surgical medicine and how further innovation is required to fully realize AI's transformative potential for patients, healthcare professionals, and practitioners. Ultimately, integrating AI into OR management holds the potential for improving patient outcomes and healthcare productivity.

## Introduction and background

Health systems are spending greater amounts of time looking for ways to increase efficiency as a result of the growing demand for excellent health care that is readily available. Many experts believe that the Operating Room (OR), which accounts for between 35% and 40% of expenses, is the financial center [1].

One of the greatest ways to characterize the OR is complexity; complicated surgical case scheduling, high patient demands, interactions between various professionals, and unpredictability are just a few of the factors that make it challenging to manage [2]. Even though efforts have been made to use industrial concepts to boost efficiency, the unique features of the OR make this application challenging [3]. With the ability to analyze vast amounts of operating block data to produce interpretative models and accurate prediction estimates, assets could be used more effectively, capital waste could be reduced, and system optimization could result in a better and safer service [4].

A subset of artificial intelligence (AI) known as machine learning (ML) makes use of algorithms that are iteratively trained to learn from vast volumes of data without explicit programming [5,6]. They can extract schemes, explain them, and develop prediction models from a variety of data sources. These machines analyze a gigantic quantity of data without becoming tired, losing focus, or making thoughtless mistakes [7].

Even in a complex setting like perioperative medicine, the use of strong analysis tools, the expansion of storage capacity, and the speed at which healthcare data is being digitized will undoubtedly be essential to improving medical care [8].

Despite the growing interest in integrating ML into OR management, there remains a need to systematically evaluate the scope, effectiveness, and limitations of existing machine learning applications in this field. This systematic review aims to synthesize current evidence on the role of ML in OR management, assessing its impact on scheduling, workflow optimization, resource allocation, and patient safety. By identifying key trends, challenges, and future directions, this review seeks to provide insights into the potential of ML in transforming surgical operations and enhancing healthcare delivery.

## Review

Methodology

Protocol

The Preferred Reporting Items for Systematic Reviews and Meta-Analyses 2020 (PRISMA) guidelines were followed in conducting this systematic review [9]. Prior to data collection, a predetermined procedure was established that outlined the goals, inclusion/exclusion criteria, and analysis techniques. The protocol was not listed in a public registry because of the exploratory nature of the review.

Eligibility Criteria

The PICOS framework was used to define the eligibility criteria for study inclusion [10]. The population (P) comprised studies focusing on OR management within healthcare settings. The intervention (I) involved the implementation of ML techniques to enhance OR operations, including scheduling optimization, workflow efficiency, staff allocation, and patient safety measures. Comparisons (C) were drawn against conventional OR management methods or non-ML-based approaches. The outcomes (O) evaluated included enhancements in operational efficiency, scheduling accuracy, cost reduction, resource utilization, and patient safety. Original research studies (S) utilizing randomized controlled trials (RCTs), observational designs (cohort, case-control, or cross-sectional studies), and retrospective analyses were considered eligible for inclusion.

Inclusion and Exclusion Criteria

Studies included in this review focus on OR management in healthcare settings, encompassing aspects such as surgical scheduling, resource allocation, workflow efficiency, and patient safety, while excluding studies unrelated to OR management or those solely addressing robotic surgery without considering OR workflow. The intervention of interest involves the application of ML techniques for optimizing OR management, including scheduling, resource utilization, and efficiency improvements, whereas studies that do not incorporate ML techniques or rely solely on non-ML-based decision-making are excluded. Comparisons are made against conventional OR management methods, such as manual scheduling and traditional workflow strategies, with studies lacking a comparative approach or failing to evaluate ML against other methods being excluded. The primary outcomes assessed include improvements in OR efficiency, scheduling accuracy, cost reduction, resource utilization, and patient safety, while studies without measurable OR-related outcomes are not considered. Eligible study designs include RCTs, observational studies (cohort, case-control, cross-sectional), retrospective analyses, and systematic reviews, with case reports, editorials, commentaries, opinion pieces, and studies lacking full methodological details being excluded. Only studies published in English are considered, excluding non-English studies without available translations. Furthermore, only full-text articles available in peer-reviewed journals and reputable conference proceedings are included, while abstract-only articles, unpublished manuscripts, or studies without full-text access are excluded.

Search Strategy

To find studies that were relevant, a thorough search was done using PubMed, EMBASE, Scopus, Web of Science, and IEEE Xplore. Keywords, Boolean operators, and Medical Subject Headings (MeSH) terms were all used in the search strategy's development. Additional publications pertinent to the review were found by manually going through the reference list of the included paper. To meet the unique needs and index words of the corresponding databases, each search string was modified. Each database's specific search strings are listed in Table 1.

**Table 1:** Search strings for five different databases

Database	Search string
PubMed	("machine learning"[Title/Abstract] OR "artificial intelligence"[Title/Abstract] OR "deep learning"[Title/Abstract] OR "neural networks"[Title/Abstract]) AND ("operating room"[Title/Abstract] OR "surgical scheduling"[Title/Abstract] OR "surgical workflow"[Title/Abstract] OR "surgical efficiency"[Title/Abstract]) AND ("optimization"[Title/Abstract] OR "resource allocation"[Title/Abstract] OR "decision support"[Title/Abstract] OR "patient safety"[Title/Abstract])
EMABSE	('machine learning'/exp OR 'artificial intelligence'/exp OR 'deep learning'/exp OR 'neural network'/exp) AND ('operating room'/exp OR 'surgical scheduling'/exp OR 'surgical workflow'/exp OR 'surgical efficiency'/exp) AND ('optimization'/exp OR 'resource allocation'/exp OR 'decision support'/exp OR 'patient safety'/exp)
Scopus	TITLE-ABS-KEY ("machine learning" OR "artificial intelligence" OR "deep learning" OR "neural networks") AND ("operating room" OR "surgical scheduling" OR "surgical workflow" OR "surgical efficiency") AND ("optimization" OR "resource allocation" OR "decision support" OR "patient safety")
Web of Science	TS=("machine learning" OR "artificial intelligence" OR "deep learning" OR "neural networks") AND ("operating room" OR "surgical scheduling" OR "surgical workflow" OR "surgical efficiency") AND ("optimization" OR "resource allocation" OR "decision support" OR "patient safety")
IEEE Xplore	("machine learning" OR "artificial intelligence" OR "deep learning" OR "neural networks") AND ("operating room" OR "surgical scheduling" OR "surgical workflow" OR "surgical efficiency") AND ("optimization" OR "resource allocation" OR "decision support" OR "patient safety")

Studies Selection

Duplicate studies were eliminated during the extraction process from several databases, and all found studies were retrieved into ENDNOTE X9.3.3 (Bld 13966). The titles and abstracts were checked for relevancy by two separate reviewers (Amam and Smam) from the contributor's list. The inclusion and exclusion criteria were used to evaluate studies, for which full-text articles were accessible. A third reviewer (tiebreaker Naam) was used to settle disagreements between the first two reviewers.

Data Extraction

Data from included studies was gathered using a standardized Microsoft® Excel Spreadsheet (Microsoft, Inc., Redmond, USA). Study parameters including author, year of publication, study design, and environment were all included in the extracted data. The population demographics, clinical characteristics, and sample size were all documented. Data on AI methodology was gathered, with particular attention paid to the kind of AI algorithm employed and the techniques utilized for testing, validation, and training. 

Risk of Bias Assessment

The Newcastle-Ottawa Scale (NOS) was utilized to evaluate the risk of bias in the included studies. Each study was categorized as having a low, moderate, or high risk of bias based on factors such as selection process bias, intervention bias, deviations from the intended intervention, missing data bias, outcome assessment bias, and reporting bias. The selection criteria were assessed using the inclusion and exclusion parameters. Performance bias was evaluated by examining the presence of a control group and the implementation of allocation concealment. Various aspects, including data handling, selective reporting, biased reporting, and full industrial sponsorship, were assigned different rankings. Reviewers conducted multiple sessions to assess eligibility constraints and ensure consistency in reporting. Any discrepancies in scoring between reviewers were resolved by a second reviewer, who re-evaluated the studies to reach a consensus.

Results

Search Results

**Figure 1:**
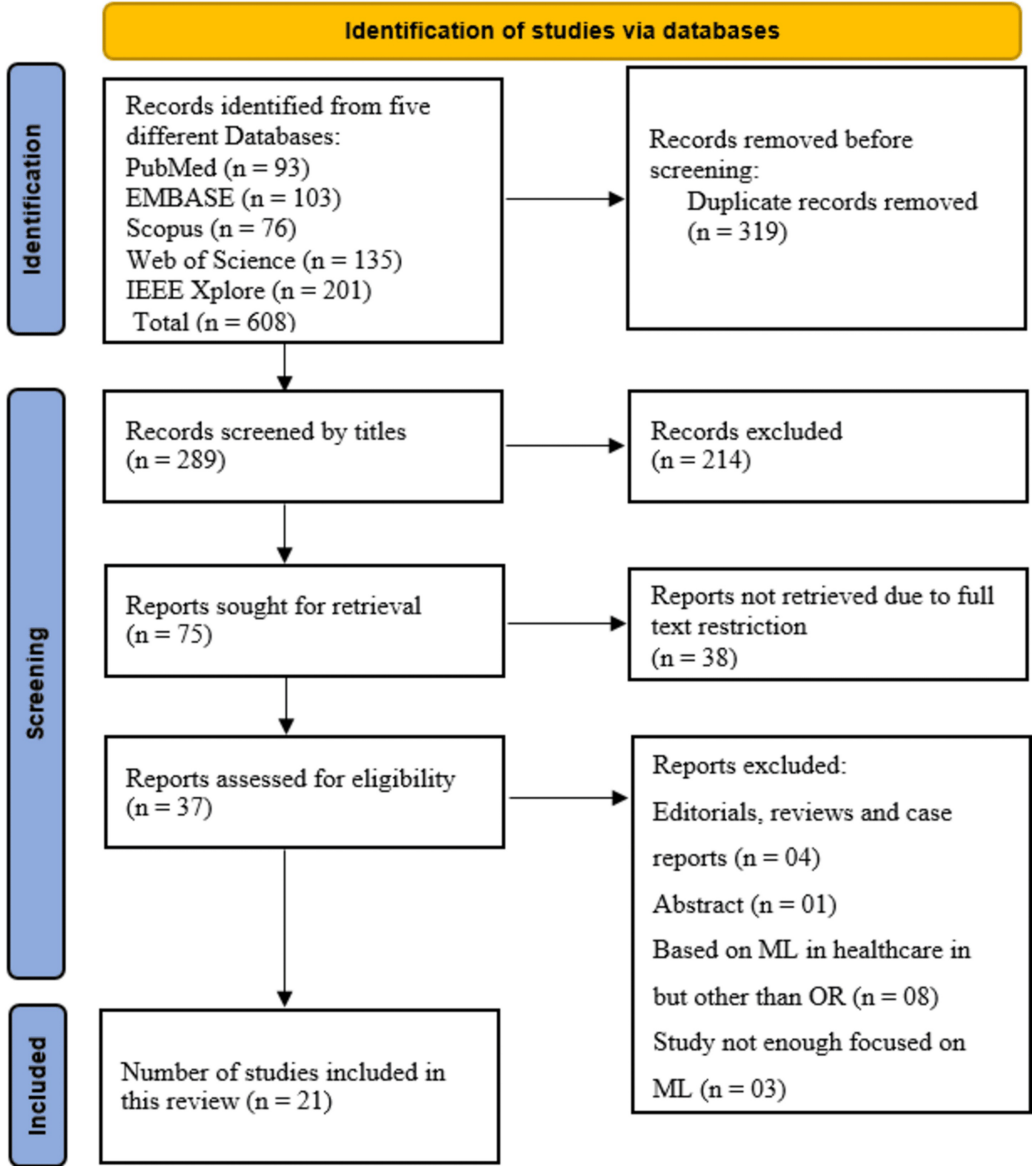
PRISMA flow diagram PRISMA: Preferred Reporting Items for Systematic Reviews and Meta-Analyses

A comprehensive search across five databases, PubMed, EMBASE, Scopus, Web of Science, and IEEE Xplore, retrieved a total of 608 records (PubMed: 93, EMBASE: 103, Scopus: 76, Web of Science: 135, and IEEE Xplore: 201). After removing 319 duplicate records, 289 unique studies remained for title screening. During the title screening phase, 214 records were excluded based on relevance, leaving 75 studies for full-text retrieval. However, 38 reports could not be accessed due to full-text restrictions, reducing the number of eligible studies to 37 for detailed evaluation. Subsequently, full-text assessment led to the exclusion of 16 studies for the following reasons: editorials, reviews, and case reports (n=4), abstract-only studies (n=1), studies on ML in healthcare but unrelated to the OR (n=8), and studies with insufficient focus on ML (n=3). Ultimately, 21 studies met all inclusion criteria and were incorporated into the systematic review. The detailed study selection process is outlined in Figure 1, following the PRISMA flowchart.

Characteristics of Included Studies

The included studies in this systematic review spanned various countries and employed different study designs to explore the application of AI in surgical settings. A total of 21 studies, published between 2019 and 2025, were analyzed. Most studies were conducted in the United States (n=11), with additional studies from China (n=02), Australia (n=02), Canada (n=01), New Zealand (n=01), Iran (n=01), Colombia (n=01, Israel (n=01), and Taiwan (n=01). All studies utilized retrospective observational designs, except for one RCT.

Sample sizes varied significantly, ranging from as small as 20 participants to as large as 124528. The primary objectives of the studies revolved around the development and evaluation of AI models for predicting surgical durations, post-anesthesia care unit (PACU), length of stay (LOS), and optimizing surgical scheduling. A diverse range of surgical procedures was covered, including otolaryngology, orthopedics, laparoscopic cholecystectomy, gynecological and colorectal surgeries, total knee arthroplasty, and spine surgeries.

Various AI methodologies were employed, including logistic regression, support vector classifiers, random forest classifiers, XGBoost, artificial neural networks, multilayer perceptron neural networks, genetic algorithms, and deep learning models such as Clinical BERT. Performance metrics varied across studies, with some reporting AUC values ranging from 0.662 to 0.82, while others measured predictive accuracy using root mean squared error (RMSE), mean absolute error (MAE), and explained variance scores. In general, ensemble learning techniques, including balanced bagging classifiers and random forest regressors, demonstrated superior predictive capabilities.

Several studies identified key predictors influencing surgical durations and PACU LOS, such as patient body mass index (BMI), sex, planned surgical case duration, and procedural complexity. Some studies also examined the role of AI in improving OR efficiency and reducing delays, showing significant reductions in PACU wait times and enhanced scheduling optimization. The findings collectively underscore the potential of AI in refining perioperative decision-making, improving workflow efficiency, and enhancing patient outcomes (Table 2).

**Table 2:** Characteristics and key outcomes of included studies ML: machine learning; PACU: post-anesthesia care unit; LOS: length of stay; OR: operating room; SMOTE: synthetic minority over-sampling technique; DCA: decision curve analysis; LASSO: least absolute shrinkage and selection operator; CI: confidence interval; BMI: body mass index; NLP: natural language processing; BERT: bidirectional encoder representations from transformers; RMSE: root mean squared error; MAE: mean absolute error; R²: coefficient of determination; MAPE: mean absolute percentage error; OT: operating theater; SHAP: shapley additive explanations; RCT: randomized control trial

Author	Publishing year	Country	Study design	Sample size	Objective	Type of procedure	Type of AI	Predictive performance	Outcomes
Gabriel et al. [11]	2022	United States	A single-centered observational retrospective study	13447	Development of ML models that forecast the following composite result: the patient was released after the conclusion of the recovery room nursing shift, and the surgery was completed by the end of the OR block period	Surgery for the ears, nose, and throat as well as orthopedics	Logistic regression, support vector classifier, balanced Random Forest classifier, Random Forest classifier, simple feedforward neural network, and balanced bagging classifier, SMOTE.	A model was developed for each of the following start times: 1 pm, 2 pm, 3 pm, and 4 pm. The results showed that the ensemble learning strategies had the best AUC scores. With F1 scores of 0.78, 0.80, 0.82, and 0.82 for forecasting the outcomes when cases began at 1 pm, 2 pm, 3 pm, and 4 pm, respectively, the balanced bagging classifier performed best	End time of surgery and PACU LOS
Tully et al. [12]	2023	United States	A single-centered observational retrospective study	10928	Development of ML models to forecast patients undergoing ambulatory surgery who may require a longer PACU stay and then model the efficacy of lowering the demand for PACU staffing after-hours	Procedures for outpatient surgery	Random Forest classifier, balanced bagging classifier, XGBoost regressor, feedforward neural network, logistic regression, and balanced random forest classifier	Extended PACU length of stay was linked to female sex (P<0.0001) and the length of the planned surgical case (P<0.0001). According to AUC, logistic regression without SMOTE performed the poorest with SMOTE (AUC 0.718), while XGBoost performed the best with SMOTE (AUC 0.779)	PACU LOS
Cao et al. [13]	2021	China	A single-centered observational retrospective study	913	Developing a predictive nomogram to help determine which LC patients are at higher risk of experiencing an extended PACU length of stay	Laparoscopic cholecystectomy	DCA, calibration plot, C-index, and LASSO regression model	This model's C-index for the training set was 0.662 (95% CI 0.603 to 0.721), and for the test set it was 0.609 (95% CI 0.549 to 0.669), indicating effective calibration and moderate discrimination. DCA showed that when a procedure was chosen at the potential threshold of 7%, an extended PACU LOS the nomogram was dependable for clinical use	PACU LOS
Schulz et al. [14]	2020	Australia	A single-centered observational retrospective study	67325	Development of case-mix or risk-adjusted LOS benchmarks for PACUs that may be incorporated into contemporary reporting systems	Each case in which a doctor uses anesthesia	MinMax scaling	A large portion of the variation in the mean PACU LOS for individual anesthetists could be explained by this predictive model (Spearman's r²=0.57). Anesthetists fell within a substantially narrower range when the projected PACU LOS was subtracted; 80% of them had mean LOSDs within a band of just 4.3 minutes, compared to a spread of 24 minutes for the uncorrected mean LOS	PACU LOS
Rozario [15]	2020	Canada	A single-centered observational retrospective study	10553	Developing personalized models to maximize the effectiveness of OR reservation times	All types of surgeries	The Google AI OR Tools software suite, which is open source, and the Python programming language	Additionally, there were 26 minutes of delays (95% CI: 25, 27 minutes), which equated to an 80% reduction in PACU admission wait times The optimized plan included 113 minutes of PACU waits, a 76% reduction.	Time optimization for operations
Strömblad et al. [16]	2021	United States	RCT	683	Evaluation of the precision and practical results of applying an ML model to forecast the length of a surgical case	Gynecological and colorectal surgery	Random Forest	When in comparison to the control group, the use of an ML model substantially increased the accuracy of predicting case duration and resulted in shorter patient wait times, no difference in time between the cases (such as turnover or surgeon wait times), and shorter presurgical lengths of stay. In the intervention arm, the MAE SD and mean error SD for colorectal treatment would have decreased from 87 to 70 and 103 to 86, respectively.	Estimation of each planned surgery's length, expressed in terms of MAE and (arithmetic) mean (SD) error
Yeo et al. [17]	2023	United States	A single-centered observational retrospective study	10021	Development of a precise surgical time prediction model for individuals having a primary total knee replacement	Total knee arthroplasty	K-Nearest neighbor, Random Forest, and artificial neural networks	The three biggest predictors of surgical operative time were a high BMI (>40 kg/m2), younger age, and non-usage of tranexamic acid. The precise estimation (AUC=0.82) is crucial for improving OR efficiency and recognizing patients at risk for extended surgical operating time	Prediction of duration of surgeries
Adams et al. [18]	2023	New Zealand	A single-centered observational retrospective study	35000	There are two ways to forecast how long a surgical procedure will take by taking into account the medical facts about the surgery	Surgical operations	Linear regression	For surgeries that are not performed frequently, the ontological information improves the continuous ranked likelihood scores of procedure time prediction from 18.4 to 17.1 minutes and from 25.3 to 21.3 minutes	Prediction of procedure durations
Zhong et al. [19]	2024	United States	A single-centered observational retrospective study	201	Use of ML and NLP to understand radiology information for patients having radius fracture repair as part of a proof-of-concept study to forecast case length	Internal repair of the radius fracture through open reduction	Baseline model, multilayer perceptron neural network, random forest regressor, linear regression, performance metrics, and k-fold cross-validation	With Clinical BERT outputs, feedforward neural networks achieved the lowest average RMSE, which was significantly (P<0.001) lower than that of the baseline model. For the test set, the proportion of correctly predicted cases, defined as the actual surgical length falling within 15% of the expected surgical duration, rose from 26.8% to 58.9% (P<0.001) when a feedforward neural network and Clinical BERT were used	Estimating the length of surgery
Miller et al. [20]	2023	United States	A single-center observational study	50888	ML procedures are improved by extending case durations over current non-ML methods for instances involving head and neck surgeries and otolaryngology	Otolaryngology surgery case	XGBoost and CatBoost	Compared to the XGBoost model, the CatBoost model showed superior predictive ability (P=0.041). Both models, however, outperformed the baseline model (P<0.001), reducing the operative period MAE by 9.6 and 8.5 minutes, respectively, compared to the current techniques	Prediction of surgery duration
Eshghali et al. [21]	2024	Iran	A single-centered observational study	20	Creation of a method for OTs to use in scheduling and rescheduling both scheduled and emergency patients	All surgeries	Particle swarm optimization, Random Forest, CPLEX, traffic congestion index, and genetic algorithm	The findings indicate that using the suggested methodology can enhance OT performance by an average of about 10.5%	Prediction of surgery duration
Gabriel et al. [22]	2023	United States	A single-centered retrospective study	3189	Using an ensemble learning strategy that could increase the precision of the spine surgery case length schedule	Spine surgery	Random Forest regressors, bagging regressors, XGBoost regressors, and multivariable linear regression	With an RMSE of 92.95 minutes, an MAE of 44.31 minutes, an explained variance score of 0.778, and an R² of 0.770, the XGBoost regressor outperformed the others. BMI, spinal combinations, surgical method, and the number of spine segments involved were the factors that had the greatest impact on the model, according to SHAP evaluation of the XGBoost regression	Case duration Prediction
Chu et al. [23]	2022	Taiwan	A single-centered observational retrospective study	124528	Developing prediction models to analyze the performance of various models and precisely forecast the OR room utilization time	All surgeries	Artificial neural network, Random Forest, XGBoost, and convolution neural network	The results of their best-performing XGBoost model were determined to be 31.6 minutes, 18.71 minutes, 0.71, 28%, and 27% for the metrics of RMSE, MAE, R², MAPE, and the percentage of the estimated result that varied by 10%, respectively. A deviation of 5 to 10 minutes would be more instructive for users in the actual application; therefore, we have included our predicted findings for each department separately	Surgical time prediction
Huang et al. [24]	2022	China	A single-centered observational study	15754	Development of a system for predicting the duration of anesthesia and operation	All surgeries	Perceptron	When the anesthetic emergence duration prediction system and the surgery duration forecast system are combined, the prediction accuracy is more than 0.95.	Prediction of surgical duration and anesthesia emergence length
Gabriel et al. [25]	2022	United States	A single-centered observational retrospective study	13447	Building ML models that forecast the following composite result: the patient is released by the end of the recovery room nurse’s shift, and the surgery is completed by the end of the OR block period	Orthopedics and ENT surgeries	Simple feedforward neural networks, balanced random forest classifiers, support vector classifiers, logistic regression, balanced random forest classifiers, and balanced bag classifiers	AUC values were highest for ensemble learning approaches, according to a model developed for each start time. With F1 scores of 0.78, 0.82, and 0.82 for forecasting the result when cases began at 1 pm, 2 pm, 3 pm, or 4 pm, respectively, the balanced bagging classifier outperformed the others	Time of surgery completion and release from the recovery room
Lam et al. [26]	2022	United States and Singapore	Double-centered observational retrospective study	7585	Assessment of the effectiveness of the ML models and current surgery case duration estimators in predicting the length of operation at two sizable tertiary healthcare facilities	Colorectal surgeries	CatBoos	In terms of RMSE, MAE, MAPE, and the percentage of cases within 80%-120% of the expected actual duration, the basic MA-based forecasts perform better than the predicted duration supplied by the OR schedulers. Model 5 performs best in center-1, with an MAE of 23.986, RMSE of 45.18, and MAPE of 34.40%. In center 2, Model 5 performs the best, with 56.11% of its forecasts falling within +/- 20% of the actual duration. The prediction accuracy of Model 5 is 7.78% greater than that of the MA (within +/-20%). Additionally, Model 5 has the lowest values for MAE and RMSE, at 23.36%, 23.61%, and 38.48%, respectively	Calculating surgery durations
Abbou et al. [27]	2022	Israel	Double-centered observational retrospective study	102103	Enhancement of OR efficiency and usefulness	All surgeries	The XGBoost model and the naïve model are based on the median duration of comparable surgeries	The XGBoost models outperformed the naïve models using various performance evaluation metrics: the MAE was 21.5 versus 25.4 in hospital 1 and 25.3 versus 28.7 in hospital 2; the RMSE was 36.6 versus 49.0 in hospital 1 and 40.3 versus 55.0 in hospital 2; the PVE was 66.7 versus 44.0 in hospital 1 and 70.0 versus 46.8 in hospital 2; and the ML2R was 0.46 versus 0.53 in hospital 1 and 0.46 versus 0.49 in hospital 2. Based on hospital performance evaluations, the naïve and ML-based models differed only slightly in the case of MAPE: 35.15 versus 35.37 at hospital 1 and 35.09 versus 32.48 at hospital 2.	Anticipated duration of stay
Hassanzadeh et al. [28]	2022	Australia	A single-centered observational study	99732	Using data from operating theaters to enhance decision-making for better theater management	Emergency and elective surgeries	Sigmoid, poly, SVM, RBF, rolling window, regression, decision tree, Random Forest, bagging regressor, gradient boosting regressor, XGBoost regressor, and ensemble regressor	One practical aspect of theater management is forecasting OR demand, which helps hospitals deliver services as effectively and efficiently as possible to achieve the greatest possible health results. They were 90% accurate in their predictions	Forecasting daily demand for surgery by medical specialty
Jiao et al. [29]	2022	United States	Multicenter-centered observational retrospective study	70826	Creation of an ML method for predicting surgical duration that continuously integrates preoperative and intraoperative data	All surgeries	Modular ANN	The modular ANN outperformed the Bayesian strategy by a large margin, with the lowest time error. In addition, the modular ANN outperformed the Bayesian strategy (80%) and a naive approach utilizing the scheduled time (78%), with the highest accuracy in detecting ORs that would exceed 15:00	Techniques for estimating the length of a procedure
Martinez et al. [30]	2021	Colombia	Single-centered observational study	81248	Enhancing the operation scheduling duty, which necessitates estimating the surgical time length, would maximize OR efficiency	One-step operations and surgeries	Support vector regression, regression trees, bagging regression trees, and linear regression	Using a subset of the database that included the nine specialties that accounted for 80% of the surgeries, Bagged trees produced the best overall performance. With a lower RMSE, bagged trees also fared better than the experience-based approach	Predicting the surgical time
Bartek et al. [31]	2019	United States	Single-centered observational retrospective study	14345	Building statistical models to enhance case-time duration estimate	All surgeries	XGBoos and Random Forest	With the use of the ML surgeon-specific approach, the ability to forecast cases within 10% increased from 32% using our institution's standard to 39%. Forty-five percent of the models had accuracy levels higher than or equal to those of the schedulers. Compared to surgeon schedulers, these algorithms significantly outperformed them, with predictions as high as 50% within 10%, versus 32%	Predicting the surgical time

Risk of Bias Assessment

The risk of bias assessment was conducted using the NOS. Among the 21 studies included, 17 were identified as having a low risk of bias, while four exhibited a moderate risk of bias. A common methodological limitation in some studies was the selection of controls. Additionally, none of the studies reported blinding of controls and patients regarding exposure, which may have introduced measurement bias. Furthermore, the GRADEpro GDT (Evidence Prime, Hamilton, Canada) assessment indicated that the overall quality of evidence in this meta-analysis was low. This was primarily due to the inclusion of observational studies (case-control), which inherently carry a higher risk of bias due to the inability to randomize exposure. Additionally, inconsistencies across studies further contributed to the low quality of evidence (Table 3).

**Table 3:** Risk of bias assessment using the NOS tool The rating scale assigns 7 to 9 stars for a low risk of bias, 4 to 6 stars for a moderate risk of bias, and 0 to 3 stars for a high risk of bias. Selection: (1) If the definition is adequate? (2) If the case representativeness is ok? (3) Controls selection (community or hospital). (4) Controls definitions. Comparability: (1) Comparability of controls and cases according to the analysis or design. Exposure: (1) Exposure determination. (2) The same method for calculation controls and cases. (3) Non-response rate. A single star (★) can be awarded to a study for each numbered item in the exhibit and selection categories. For comparability, no more than two stars (★★) can be given. Hyphen (-) indicates no stars were given to the study. NOS: Newcastle-Ottawa scale

Study	Selection				Comparability	Exposure		
	1	2	3	4	1	1	2	3
Gabriel et al. [11]	★	★	★	-	★★	★	★	-
Tully et al. [12]	★	★	-	-	★★	★	-	-
Cao et al. [13]	★	★	-	★	★	★	★	-
Schulz et al. [14]	★	★	-	-	★★	★	★	-
Rozario [15]	★	★	★	-	★	-	★	★
Strömblad et al. [16]	★	★	-	-	★★	★	★	★
Yeo et al. [17]	★	★	★	-	★★	★	★	★
Adams et al. [18]	★	★	-	-	★★	★	★	★
Zhong et al. [19]	★	★	★	-	★★	★	★	★
Miller et al. [20]	★	★	★	-	★	★	★	★
Eshghali et al. [21]	★	★	★	-	★★	★	-	★
Gabriel et al. [22]	★	★	-	-	★★	★	★	★
Chu et al. [23]	★	★	★	-	★	★	★	★
Huang et al. [24]	★	★	-	-	★★	★	★	★
Gabriel et al. [25]	★	★	★	-	★	★	★	★
Lam et al. [26]	★	★	★	-	★★	★	-	★
Abbou et al. [27]	★	★	-	-	★★	★	★	★
Hassanzadeh et al. [28]	★	★	★	-	★	★	★	★
Jiao et al. [29]	★	★	-	-	★★	★	★	★
Martinez et al. [30]	★	★	★	-	★	★	★	★
Bartek et al. [31]	★	★	-	-	★★	★	★	★

Discussion

The findings of this systematic review highlight the growing role of ML in optimizing OR management, particularly in predicting surgical durations, PACU LOS, and scheduling efficiency. The included studies collectively demonstrate that ML models, leveraging a range of methodologies, can enhance decision-making processes, streamline OR workflows, and improve patient outcomes. These results align with broader literature emphasizing the transformative impact of AI in perioperative care [32-34].

Predicting the length of surgical planning was the focus of 17 of the 21 included studies. This finding emphasizes how important precise estimation of surgical case length is to efficient OR management. It is a complicated and multidimensional problem that has a significant effect on resource allocation, OR scheduling, and overall operational effectiveness. The main focus of one prior review was the encouraging outcomes of a proprietary algorithm called Leap Rail® [3,35]. A recent study shows a more nuanced picture, even if it demonstrated an improvement in predicting accuracy when compared to previous methods [30]. The application of ML models has been examined in greater detail in more recent research, such as that done by Bartek et al., which highlights the significance of surgeon-specific models [31].

The ML recent models perform better than those tailored to a particular service and greatly improve case-time forecast accuracy, which has major advantages for OR administration [31]. A recent study shows that XGBoost outperforms other methods, such as linear regression and the random forest model, in ML models [36]. A significant departure from the previous review's emphasis on leap rail® is the demonstration of XGBoost's stronger predictive capabilities. This demonstrates how quickly ML technology is developing and how it may be used to improve surgical case duration forecasts. It's crucial to remember, though, that various results can call for various ML techniques [37].

The potential cost savings linked to precise surgical case duration estimations in robotic surgery was another important conclusion from the earlier review [3]. Our review, however, offers fresh perspectives. The application of modular ANN for estimating the remaining surgery duration was first presented by Jiao et al. [29]. Neural networks with external memory are called modular ANN [38]. They do well on activities that call for sequential reasoning and context, which qualifies them for several clinical applications [28]. The study demonstrated the resilience and versatility of their strategy by using anesthetic records from a variety of surgical populations and facility types. Modular ANN has the potential to reduce costs and enhance operational efficiency because it continuously outperformed Bayesian statistical techniques, especially in the final quartile of surgery [5]. Additionally, a study evaluated the modular ANN model's transferability and generalizability [27]. It was discovered that optimizing a model developed at larger adjacent systems could help even healthcare systems with lower operational volumes. Additionally, it indicated areas for improvement by highlighting the dearth of vital data in the anesthesia records during specific surgical phases [11,26]. The quick development of ML algorithms and their use in actual surgical situations is highlighted by this work.

This is likewise the case for variational autoencoders (VAEs), generative models that are intended to learn latent representations of data [39]. They include a decoder and an encoder. In a latent space, for example, the encoder converts input data into a probability distribution, and the decoder uses samples in this latent space to reconstruct the data. Connecting cutting-edge models such as modular VAEs and ANNs to a clinical context suggests that these models may help advance personalized healthcare by learning illustrations unique to each patient, facilitating customized treatment regimens and meeting clinical requirements, improving diagnostics and patient outcomes, or expediting medical procedures [17,19,23,24].

Additional information was provided by the single-center, RCT carried out by Strömblad et al. [16]. In contrast to the current scheduling-flow approach, they investigated the precision of utilizing an ML model to forecast surgical case durations. The advantages of a thorough and data-driven prediction method were highlighted in this study, which led to a notable decrease in MAE and improved forecast accuracy [15]. Significantly, this drop in MAE resulted in shorter wait times for patients without negatively impacting wait times for surgeries or operational effectiveness, suggesting a delicate balance between effectiveness and patient outcomes. To the best of our knowledge, this study is the very first and sole RCT on the topic, marking an important milestone [16].

The results of this review are consistent with previous research demonstrating the utility of ML in perioperative settings. For instance, Gabriel et al. (2022) found that ensemble learning models, including balanced bagging classifiers, exhibited high predictive accuracy (AUC up to 0.82) in forecasting PACU LOS and end-of-surgery times [11]. This finding aligns with similar studies that have identified ensemble techniques, particularly random forest classifiers, as robust predictors in clinical applications. A systematic review by Jiao et al. (2023) on AI-driven surgical predictions also noted that ensemble methods tend to outperform individual models due to their ability to mitigate bias and variance, further reinforcing the efficacy of such approaches [29].

Moreover, studies such as Tully et al. identified key predictors influencing PACU LOS, including sex and planned surgical case duration, which is in agreement with previous investigations highlighting patient-specific and procedural factors as critical determinants of postoperative recovery times [12]. Similarly, Strömblad et al. reported that ML models significantly enhanced case duration predictions, leading to reduced patient wait times and improved scheduling accuracy [16]. These findings resonate with those of Chan et al., who demonstrated that ML-enhanced ontological data improved surgical duration predictions, further solidifying the role of AI-driven analytics in procedural planning [40].

The reviewed studies underscore the potential of ML in optimizing OR efficiency through predictive modeling. Several studies demonstrated that ML models can accurately forecast surgical duration, which is crucial for reducing inefficiencies such as idle OR time, delays, and scheduling conflicts. For example, Yeo et al. (2023) reported that high BMI, younger age, and the absence of tranexamic acid were key predictors of prolonged surgical times [17]. This finding suggests that integrating patient-specific variables into predictive models can yield clinically meaningful insights, ultimately improving resource allocation and perioperative planning.

Additionally, the use of ML in reducing PACU wait times presents a compelling case for AI-driven hospital management. Rozario observed that AI-based OR scheduling optimizations resulted in an 80% reduction in PACU admission wait times [15]. This supports the growing body of evidence advocating for AI-enhanced workflow management systems to alleviate bottlenecks in perioperative care.

Despite these promising results, the variability in ML model performance across studies warrants careful consideration. While some studies achieved high predictive accuracy (e.g., AUC >0.80 in Gabriel et al. [11] and Yeo et al. [17]), others reported moderate predictive capabilities (e.g., Cao et al. [13] with a C-index of 0.662). Such discrepancies may stem from differences in sample sizes, feature selection methodologies, or ML algorithms used. Furthermore, the reliance on retrospective data in most studies limits the generalizability of findings, highlighting the need for prospective validation and real-time implementation trials.

Limitation

This review has some limitations, including potential publication bias since only English-language articles were considered. Differences in database coverage may also affect comprehensiveness, despite efforts to minimize this through a rigorous search strategy. The diversity of settings and algorithms complicates definitive conclusions on the optimal predictive model for perioperative complications. Lack of standardization across studies hindered meta-analysis, and most models lacked external validation. While AUC is a useful evaluation metric, its limitations in imbalanced datasets must be acknowledged. Ensuring high-quality data is crucial for AI applications in research, clinical practice, and healthcare systems, requiring careful oversight from data collection to model selection.

## Conclusions

This systematic review highlights the expanding role of ML in optimizing OR management, demonstrating its potential to enhance efficiency, cost-effectiveness, and patient safety. AI-driven models have shown promise in predicting surgical durations with greater accuracy, improving resource allocation, and minimizing case cancellations, thereby streamlining workflow coordination and reducing operational inefficiencies. These advancements not only improve hospital productivity but also contribute to better patient experiences by minimizing delays and optimizing perioperative care. However, despite these promising developments, several challenges must be addressed before AI can be seamlessly integrated into routine surgical planning and decision-making. Limited access to high-quality, standardized datasets remains a major hurdle, as does ensuring data privacy and security in compliance with healthcare regulations. Additionally, the complexity of validating AI algorithms in diverse clinical settings poses significant challenges, requiring extensive testing and external validation to ensure reliability and generalizability. Ethical considerations, such as algorithmic transparency and potential biases in AI models, must also be carefully managed to maintain trust and equity in healthcare delivery. As AI continues to evolve, its integration into clinical workflows will necessitate a balance between technological innovation and real-world applicability. The refinement of AI applications may slow publication rates as researchers prioritize robustness over rapid advancements, emphasizing the need for high-quality, reproducible studies. Moving forward, continued interdisciplinary collaboration among data scientists, healthcare professionals, and policymakers will be crucial in overcoming these barriers. By fostering innovation while addressing challenges, AI has the potential to revolutionize OR management, ultimately improving healthcare delivery, optimizing resource utilization, and enhancing patient outcomes.
